# Kahweol Exerts Skin Moisturizing Activities by Upregulating STAT1 Activity

**DOI:** 10.3390/ijms22168864

**Published:** 2021-08-18

**Authors:** Hongxi Chen, Mohammad Amjad Hossain, Jong-Hoon Kim, Jae Youl Cho

**Affiliations:** 1 Department of Integrative Biotechnology, and Biomedical Institute for Convergence at SKKU (BICS), Sungkyunkwan University, Suwon 16419, Korea; chenhx19@gmail.com; 2 Department of Veterinary Physiology, College of Medicine, Chonbuk National University, Iksan 54596, Korea; mamjadh2@gmail.com

**Keywords:** kahweol, moisturizing factors, skin, STAT1

## Abstract

Kahweol is a diterpene present in coffee. Until now, several studies have shown that kahweol has anti-inflammatory and anti-angiogenic functions. Due to the limited research available about skin protection, this study aims to discern the potential abilities of kahweol and the possible regulation targets. First, the cytotoxicity of kahweol was checked by 3-4-5-dimethylthiazol-2-yl)-2-5-diphenyltetrazolium bromide assay, while 2,20-azino-bis (3ethylbenzothiazoline-6-sulphonic acid) diammonium salt and 1-diphenyl-2-picryl-hydrazyl were used to examine the radical scavenging ability. Polymerase chain reaction analysis was performed to explore the proper time points and doses affecting skin hydration and barrier-related genes. Luciferase assay and Western blotting were used to explore the possible transcription factors. Finally, fludarabine (a STAT1 inhibitor) was chosen to discern the relationship between skin-moisturizing factors and STAT1. We found that HaCaT cells experienced no toxicity from kahweol, and kahweol displayed moderate radical scavenging ability. Moreover, kahweol increased the outcome of *HAS1*, *HAS2*, *occludin*, and *TGM-1* from six hours in a dose-dependent manner as well as the activation of STAT1 from six hours. Additionally, kahweol recovered the suppression of *HAS2*, STAT1-mediated luciferase activity, and HA secretion, which was all downregulated by fludarabine. In this study, we demonstrated that kahweol promotes skin-moisturizing activities by upregulating STAT1.

## 1. Introduction

Skin is a basic and complex barrier as the first protective wall of the human body, which includes tight junctions and the stratum corneum [1,2]. There are two ways to prevent water loss from the stratum corneum: one is to ensure an abundant water level, such as by using hyaluronic acid (HA), also known as hyaluronan, and the other is to form strong cross-links, such as by using transglutaminase 1 (*TGM-1*), *occludin*, and *claudin* between the structural proteins. HA synthase (HAS) is responsible for HA synthesis in skin moisturizing. Three types of HAS (*HAS1*, *HAS2*, and *HAS3*) control three different lengths of HA [3]. Among them, the expression level of *HAS2* is approximately 900 times higher than those of other HAS isoforms, suggesting that *HAS2* plays a significant role in hyaluronan secretion [4]. The obvious downregulation of *HAS1* and *HAS3* results in skin damage, such as juvenile hyaline fibromatosis [5]. Besides these, hydrolysis of hyaluronan is also a major way to weaken hyaluronan viscosity. There are five different hyaluronidases (HYALs) in humans: HYAL-1, -2, -3, -4, and -5. Although hyaluronidases are applied in anesthetics for medical purposes [6], they are harmful to the skin when applied in excess. There is a positive correlation between HYAL and HA: the lower HYAL concentration, the higher induction of HAS and HA [7]. *TGM-1* is also a very important protein with structural function in skin cells, and it plays a key role in preventing water loss. The cornified cell envelope is a wall protecting the body from the outside environment. Mutation of the *TGM-1* gene leads to a lack of *TGM-1* enzyme, which results in a type of congenital ichthyosis known as mellar ichthyosis [8,9].

Nowadays, coffee is one of the most commonly consumed drinks around the world. Coffee’s most famous function is its anti-fatigue effect; additionally, however, other research has documented an inverse association between coffee consumption and Parkinson’s disease morbidity [10] as well as the incidence of Alzheimer’s disease [11]. With moderate coffee consumption, the possibility of coronary heart disease might also be reduced [12]. In fact, various compounds, like caffeine, antioxidants, and diterpenes, are naturally present in coffee. Cafestol and kahweol are the majority diterpenes found in coffee oil, and there is ~4 mg of diterpenes present in unfiltered coffee [13]. Cafestol and kahweol are sensitive to acids, heat, and light. Meanwhile, kahweol is highly unstable in purified form. Previously, mixtures of both have been studied traditionally. Nowadays, using advanced purification and extraction technology, kahweol alone demonstrated anti-angiogenic properties [14], inhibition of osteoclast differentiation [15], amelioration of liver inflammation [16], suppression of macrophage-mediated inflammation [17], increase of anti-oxidative properties [18], stimulation of autophagy [18], and the induction of apoptosis in human lung adenocarcinoma [19]. However, few studies about its role in skin protection, especially skin moisturization, exist. The present study therefore aims to explore the possible functions of kahweol in skin protection, especially in preventing water loss from the skin stratum corneum, as well as its putative regulation mechanisms (see Figure 2A for schematic study design).

## 2. Results

### 2.1. Skin-Protective Effect of Kahweol

**Figure 1:**
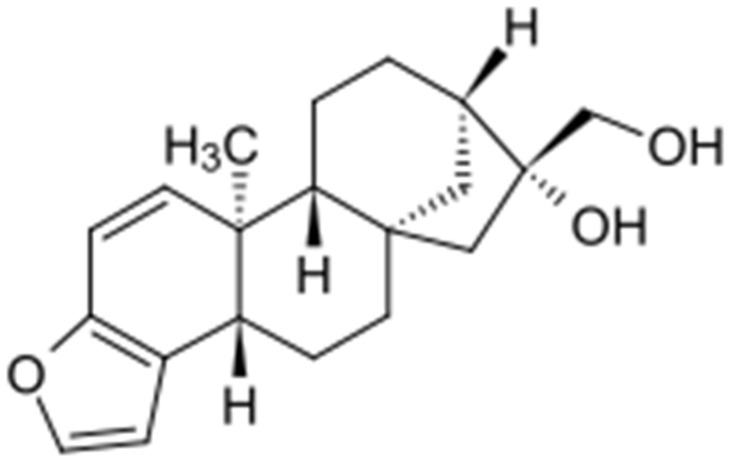
The structure of kahweol.

Kahweol, derived from *Coffea arabica* beans, is a diterpenoid molecule. It is relevant to cafestol on a structural level, as the structure of the latter lacks an extra double bond (Figure 1).

**Figure 2:**
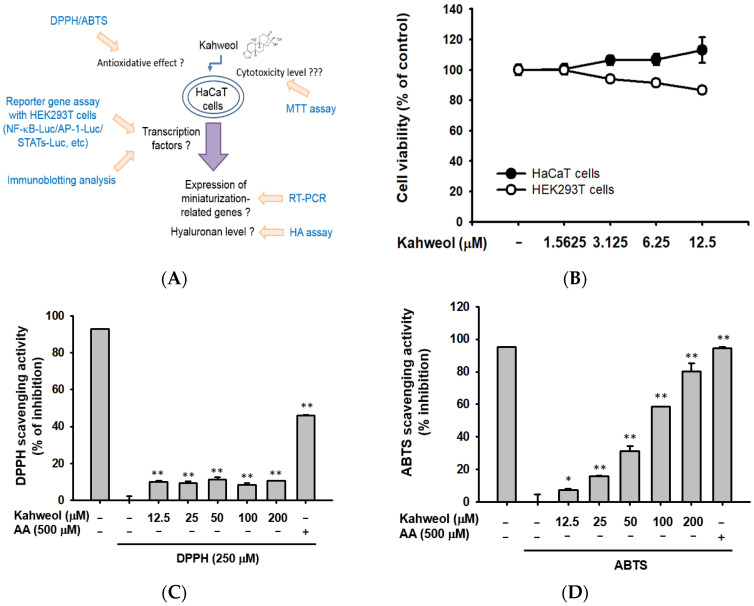

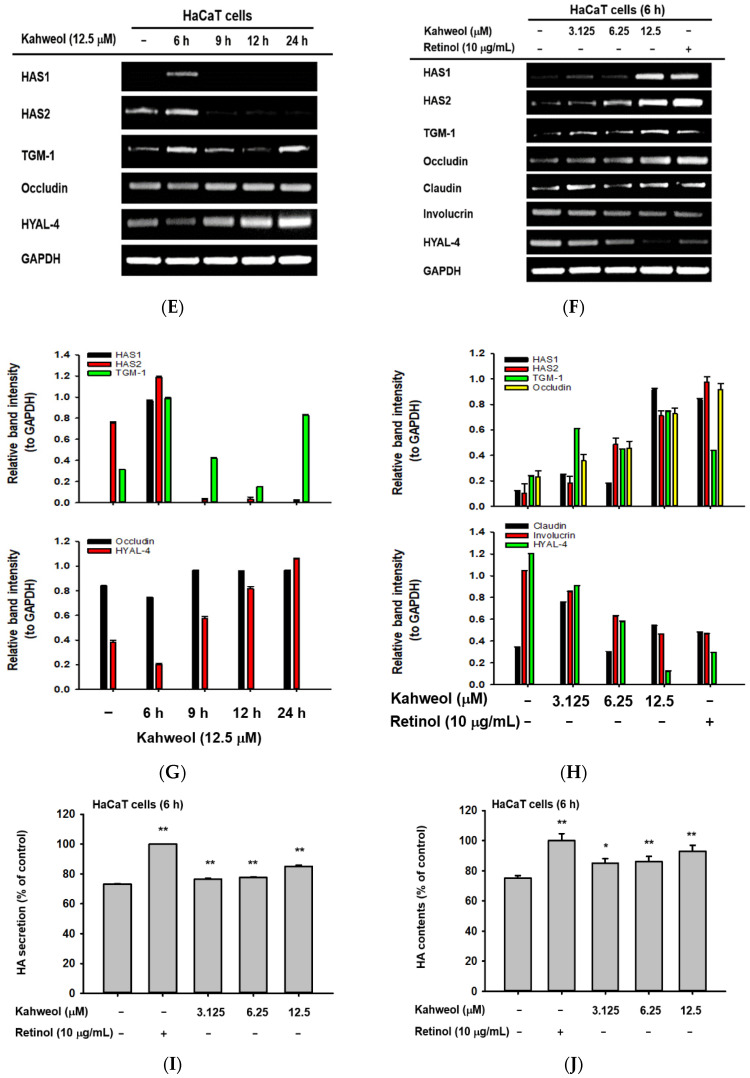
Skin-protective effects of kahweol. (**A**) Schematic design of this study. (**B**) Viability of HaCaT cells and HEK293T cells treated with different concentrations of kahweol was evaluated by MTT assay. (**C**,**D**) The radical-scavenging effects of kahweol (12.5, 25, 50, 100, and 200 μM) and AA (500 μM) were assessed by (**C**) DPPH assay and (**D**) ABTS assay. (**E**,**F**) After treatment of kahweol with indicated concentrations, HaCaT cells were collected at different time points. Then, the expression levels of *HAS1*, *HAS2*, *TGM-1*, *occludin*, *claudin*, *involucrin* and *HYAL-4* were measured by RT-PCR. (**G**,**H**) The relative intensities of mRNA levels of *HAS1*, *HAS2*, *TGM-1*, *occludin*, *claudin*, *involucrin* and *HYAL-4* were measured by ImageJ. (**I**,**J**) After treatment with 12.5 μM of kahweol for 6 h in HaCaT cells, the (**I**) secretion and (**J**) contents of HA were measured by HA assay. * *p* < 0.05 and ** *p* < 0.01 compared to normal group.

The cytotoxicity of kahweol was evaluated by setting four different doses (1.5625, 3.125, 6.25, and 12.5 μM) using the 3-4-5-dimethylthiazol-2-yl)-2-5-diphenyltetrazolium bromide (MTT) assay. Until 12.5 μM, cell viabilities were ~110% in HaCaT cells, which demonstrated that kahweol showed no harm to human skin cells (Figure 2B). In order to find the antioxidant effects of kahweol, 2,20-azino-bis (3ethylbenzothiazoline-6-sulphonic acid) diammonium salt (ABTS) and 1-diphenyl-2-picryl-hydrazyl (DPPH) assays were performed. In this experiment, ascorbic acid (AA) was chosen to be the positive control given its high capacity for radical scavenging [20]. In the DPPH assay, kahweol showed DPPH scavenging activity from 12.5 μM, or ~10%, while AA showed a greater ability to clear DPPH of almost 50% (Figure 2C). In the ABTS assay, ABTS was cleared by kahweol in a dose-dependent manner (Figure 2D). Especially at 200 μM, the scavenging influence was largely the same as that of the AA group. Additionally, the half-maximal inhibitory concentration (IC_50_) values were > 200 μM and 84.45 μM in the DPPH and ABTS assay, respectively. These data suggest the possible ability of kahweol in clearing radicals. To explore the skin-protective abilities of kahweol in HaCaT cells, several genes in charge of moisturizing conditions, like skin hydration efficacy relevant gene (*HAS1* and *HAS2*), skin barrier-associated molecules (*FLG*, *TGM-1*, *claudin*, *involucrin*, and *occludin*), and skin hyaluronan-degrading hyaluronidase 4 (*HYAL-4*), were measured at the RNA level. Retinol, also known as vitamin A1, was chosen to be a positive control as retinol leads to the high expression of HA. HA is the key molecule linked to wrinkle formation reduction and skin moisturization [21]. First, we chose 12.5 μM to test time points. As Figure 2E,G shows, the expression levels of *HAS1*, *HAS2*, and *TGM-1* were strongly increased after treatment with kahweol for six hours, meaning that their expression levels were induced to a peak within six hours. The outcome of *HYAL-4* during kahweol-treated conditions was lower than normal groups only at 6 h. Consequently, different doses of kahweol were then examined under six-hour induction conditions. As shown in Figure 2F,H, the expression levels of *HAS1*, *HAS2*, *occludin*, and *TGM-1* in the retinol group were dramatically higher than those in the normal group. What is more, kahweol dose-dependently upregulated the expression levels of *HAS1*, *HAS2*, *occludin*, and *TGM-1*, especially at 12.5 μM. As *HYAL-4*, which in charge of degrading hyaluronan, was inhibited by kahweol in dose-dependent manner (Figure 2F,H), the levels of secreted HA into media and its cellular contents were also examined. Figure 2I,J showed that kahweol is able to promote HA production both in secretion and contents.

### 2.2. Effect of Kahweol on Transcription Factor Regulation

**Figure 3:**
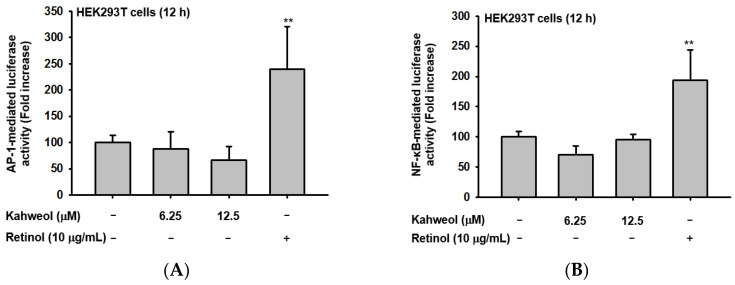

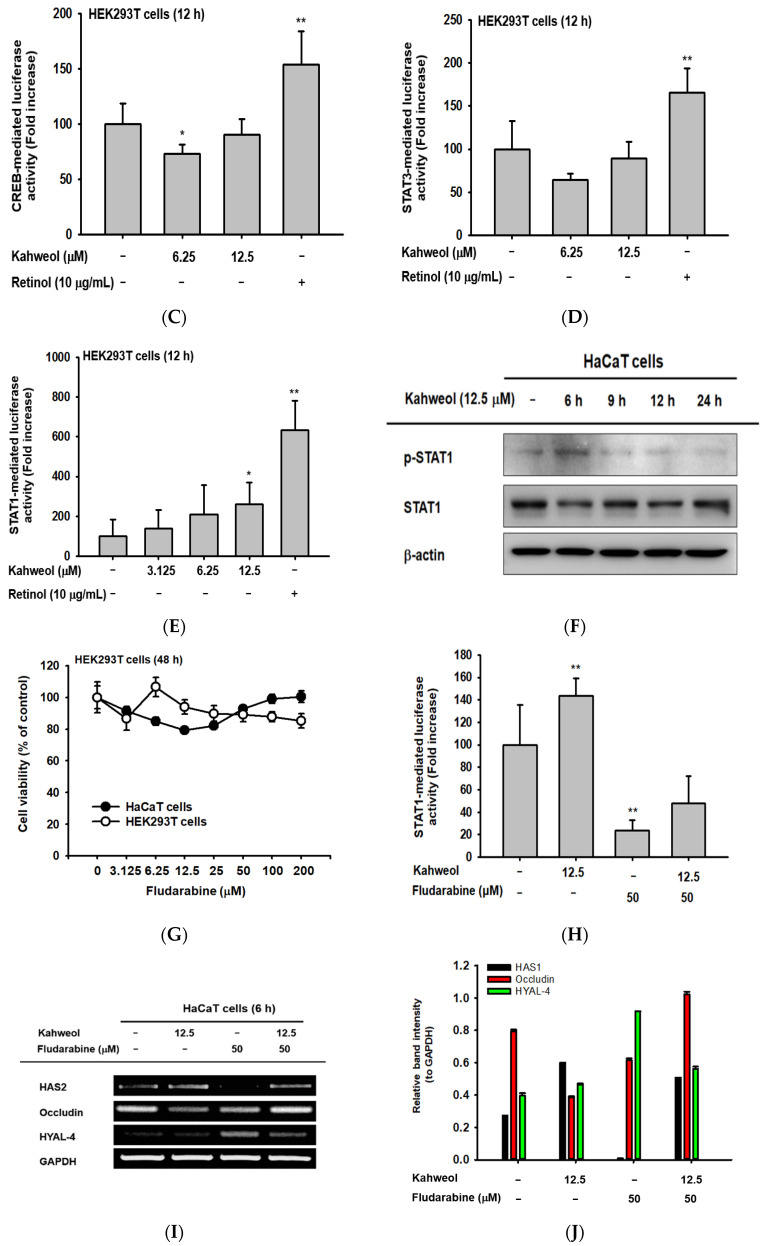

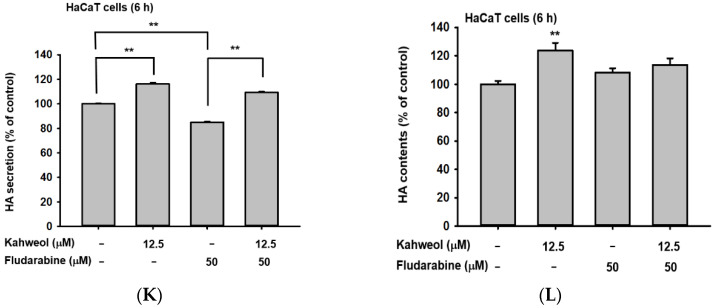
Effect of kahweol on transcription factor regulation. (**A**–**E**) AP-1–, NF-κB–, CREB-, STAT3-, and STAT1-mediated luciferase activities in HEK293T cells were measured by reporter gene assay with an illuminometer. (**F**) The occludin- and total protein levels of STAT1 and β-actin in whole-cell lysates were measured in kahweol-treated HaCaT cells. (**G**) The cytotoxicity of fludarabine in HaCaT cells and HEK293T cells was evaluated by MTT assay. (**H**) After pretreatment with fludarabine for 30 min in HEK293T cells, kahweol was then administered for a further 12 h to HEK293T cells. STAT1-mediated luciferase activity was then measured by reporter gene assay using an illuminometer. (**I**,**J**)The mRNA levels of *HAS2*, *occluding*, and *HYAL-4* in kahweol-treated HaCaT cells pre-treated with fludarabine were determined by RT-PCR (**I**). The relative intensity of mRNA levels of *HAS2*, *occluding*, and *HYAL-4* was measured by ImageJ. (**K**,**L**) After 30 min of fludarabine pretreatment, kahweol was then given for further 6 h, supernatant media was collected to detect HA secretion (**K**), and HaCaT cells were harvested to detect cellular HA contents (**L**). * *p* < 0.05 and ** *p* < 0.01 compared to the normal group.

*HAS1* and *HAS2* are known to be regulated by several signaling pathways, such as AP-1, NF-κB, CREB, STAT3, and STAT1 [22]. Therefore, five transcriptional factor-mediated luciferase assays were performed to explore the potential pathways regulated by kahweol treated for 12 h. Interestingly, retinol upregulated all five transcriptional factors (AP-1, NF-κB, CREB, STAT3, and STAT1) (Figure 3A–E). However, kahweol failed to increase four transcriptional factor-mediated luciferase activities excepting STAT1 (Figure 3E). Kahweol increased STAT1 luciferase activity in a dose-dependent manner, particularly in the 12.5 μM group. Thus, we directly checked the expression of STAT1 at the protein level with kahweol treatment at different time points. Similarly, among four different time points, the expression of p-STAT1 was clearly increased only at six hours, which suggested that kahweol modulated STAT1 in an early time frame (Figure 3F). To further confirm whether STAT1 was the target influenced by kahweol, a specific inhibitor of STAT1, fludarabine, was used to conduct a luciferase assay. Fludarabine, a nucleoside analog, achieves inhibition of STAT1 activation induced by cytokines as well as STAT1-dependent gene transcription [23]. After checking MTT, fludarabine showed no cytotoxicity in both HaCaT and HEK293T cell lines up to 48 h (Figure 3G). As we expected, fludarabine largely blocked STAT1 activity, but the suppression caused by fludarabine was reversed by kahweol (Figure 3H), which supported the previous Western blot results. To explore the regulation mechanism of kahweol between moisturizing factors (*HAS1*, *HAS2*, and *TGM-1*) and STAT1, we also used fludarabine to conduct polymerase chain reaction (PCR). As seen in Figure 3I,J, the outcome of *HAS2* was higher in the kahweol group, while it was strongly blocked due to fludarabine administration. Obviously, with the presence of fludarabine, the *HYAL-4* expression level was almost two-fold higher than that in normal group, while this increase was significantly blocked due to kahweol pretreatment. After pretreatment with kahweol for 30 min, the expression level of *HAS2* and *occludin* recovered, despite the presence of fludarabine, which suggested that kahweol regulates *HAS2* and *occludin* through STAT1. Furthermore, the secretion of HA became less than that in normal group after treating fludarabine, and kahweol upregulated this decrease after 6 h treatment (Figure 3K). However, HA contents showed no difference between fludarabine group and kahweol/fludarabine-co-treatment group (Figure 3L). These data suggested kahweol played a more significant role in promoting HA secretion instead of increase of its contents in the cells through STAT1 regulation.

## 3. Discussion

Skin aging is a biological process, including both intrinsic and extrinsic aging. Though these two types of aging are independent processes, there are many similar molecular mechanisms between them [24]. First, reactive oxygen species (ROS) are responsible for both extrinsic or intrinsic skin aging [25]. Therefore, we checked on the radical scavenging ability of kahweol by using ABTS and DPPH assays. Ultimately, the radical scavenging ability of kahweol in DPPH assay was weaker than that of AA, while this was the opposite in the ABTS assay. Figure 2C,D presents IC_50_ values of1141 μM and 84.45 μM in DPPH and ABTS assays, respectively.

Second, a lack of water in the skin plays a major role in skin aging. The characteristic molecule in this context is HA, synthesized by HAS. HA is the majority component of skin layer, existing in the extracellular matrix. HA is associated with a variety of functions, like hydration, joint lubrication, and the ability of filling space [26]. Thus, we examined three different types of HAS; however, only *HAS1* and *HAS2* were induced by kahweol. This compound most significantly affected gene expression early on (six hours) relative to at other time points (Figure 2E,G), suggesting its impact is quick to occur but not persistent. In fact, the dynamic turnover rate of HA is short, and the half-life is one day within the skin [27]. Under ultraviolet B irradiation conditions, the expression peaks of *HAS2* occurred at eight and 36 h [28]. Under six-hour treatment conditions, the proper doses of kahweol were checked by PCR as well. Using 12.5 μM of kahweol, the expression of skin hydration and barrier genes was largely induced, especially *HAS1* and *HAS2*, whose expression was almost similar to that in the retinol group (Figure 2E,G). Correspondingly, *HYAL-4* expression was blocked as well (Figure 2E,F). Moreover, the production of HA was also higher when kahweol was treated, regardless of secretion or contents (Figure 2I,J). It was reported that skin dryness was largely decreased in dorsal skin through HA oral injection to hairless mice under UV conditions [29]. In clinical study, skin hydration and elasticity as well as stratum corneum water content was dramatically increased after hyaluronan solution oral administration [30,31]. With stronger pursuit of beauty, HA is largely used in surgical techniques to prevent facial aging, especially in lower areas of the face [32].

HAS is regulated by various signaling pathways. Lee et al. reported that HA production is increased via the EGFR and AMPK signaling pathways [33]. Additionally, skin barrier function and hydration are affected by Src/AKT/NF-κB and MAPK signaling [34,35]. The proximal promoter of *HAS2* has transcription factor-binding sites for the JAK2/STAT3 pathway [36]. Rauhala et al. found that the active purinergic P2Y2 receptor largely promotes *HAS2* expression and several signaling pathways are associated with *HAS2* regulation, such as Ca^2+^/calmodulin-dependent protein kinase II (CaMKII), calcium-activated PKC, calcium response element-binding protein (CREB), and MAP kinase cascades [28]. Saavalainen et al. observed that the novel STAT-RE played a role in activating EGFR on the *HAS2* promoter [37]. Monslow et al. identified that Sp1 and Sp3 control constitutive *HAS2* transcription [38]. After analyzing several transcriptional factors by luciferase reporter assay, we finally found that STAT1 might be the target of kahweol (Figure 3E). Subsequently, Western blot analysis (Figure 3F) confirmed that kahweol had an influence on STAT1 upregulation at six hours as well. What is more, we used fludarabine to verify that the suppression of STAT1 luciferase activity caused by this drug was recovered after treatment with kahweol (Figure 3H). These three experiments, in fact, support a possibility that STAT1 was the target during the modulation of kahweol. Additionally, we also demonstrated that the upregulation of *HAS2* and *occludin* mRNA levels, and HA secretion as well as downregulation of *HYAL*-*4* in the presence of kahweol, were restored by fludarabine, implying that STAT1 might play a central role in these events (Figure 3H–L). Interestingly, as an anti-inflammatory component, kahweol triggered the suppression of nitric oxide and prostaglandin E_2_ through the NF-κB/STAT-1 pathway [17]. This fact is consistent with our findings. What is more, kahweol showed inhibition of the tumor necrosis factor α-induced JAK2–PI3K pathway [39]. As we know, JAK2 is an upstream kinase of STAT1 [40]. Many cytokines, including interleukin (*IL)-2*, *IL3*, *IL-12*, and *IL-22*, finish signal transduction through the JAK–STAT axis [41]. These studies showed that kahweol may have a possible effect on JAK2 as well. However, whether JAK2 is involved in kahweol regulation requires further exploration.

## 4. Materials and Methods

### 4.1. Materials and Drug Preparation

Kahweol was purchased from Santa Cruz Biotechnology, Inc. (Dallas, TX, USA). Kahweol powder was dissolved with dimethyl sulfoxide to create a stock solution (100 mM). The HEK293T and HaCaT cell lines were purchased from the American Type Culture Collection (Manassas, VA, USA). Dulbecco’s modified Eagle’s medium (DMEM), fetal bovine serum (FBS), phosphate-buffered saline (PBS), reduced serum medium (Opti-MEM), and TRIzol reagent were acquired from Gibco (Grand Island, NY, USA). MTT, retinol, dimethyl sulfoxide, ABTS, polyethyleneimine, DPPH, and Borax were acquired from Sigma-Aldrich Corporation (St. Louis, MO, USA). Fludarabine was purchased from Selleckchem (Houston, TX, USA). Carbazole, H_2_SO_4_, and ethanol were obtained from DAEJUNG (Seoul, Korea). Antibodies specific for β-actin, rabbit immunoglobulin G, mouse immunoglobulin G, and the phosphorylated and total forms of STAT1 were acquired from Cell Signaling Technology (Danvers, MA, USA).

### 4.2. Cell Culture

HaCaT cells (a human keratinocyte cell line) were cultured in DMEM combined with 1% antibiotics (penicillin and streptomycin) and 10% FBS. HEK293T cells (a human embryonic kidney cell line) were cultured in DMEM combined with 1% antibiotics and 5% FBS. All cell lines were cultured at 37 °C in a 5% CO_2_ incubator.

### 4.3. Cell Viability Assay

To evaluate the cytotoxicity of kahweol and fludarabine, HaCaT and HEK293T cells were seeded into 96-well plates at 2 × 10^4^ cells/well and 6 × 10^4^ cells/well, respectively, using corresponding culture medium with different doses of kahweol or fludarabine. Cell viability was tested with the MTT assay.

### 4.4. DPPH Decolorimetric Assay

To determine the oxidant scavenging ability of kahweol, a DPPH decolorimetric assay was performed as previously reported [42]. The DPPH scavenging activity was calculated as a percentage using the following Equation (1):

DPPH scavenging effect (%) = (A_0_ − A_1_)/A_0_ × 100 (1)

where A_0_ is the absorbance of DPPH alone and A_1_ is the absorbance of the sample (kahweol or AA).

### 4.5. ABTS Assay

To determine the oxidant scavenging ability of kahweol, an ABTS scavenging assay was performed as previously reported [42]. The ABTS scavenging activity was calculated as a percentage using the following Equation (2):

ABTS scavenging effect (%) = (A_0_ − A_1_)/A_0_ × 100 (2)

where A_0_ is the absorbance of ABTS alone and A_1_ is the absorbance of the sample (kahweol or AA).

### 4.6. Luciferase Reporter Gene Assay

HEK293T cells were plated in 1.25 × 10^5^ cells/well density in 24-well plates. Then, plasmids expressing β-galactosidase, AP-1-, NF-κB-, CREB-, STAT3-, and STAT1-luciferase and polyethyleneimine were transfected into cells for 36 h. Then, cells were treated with 3.125, 6.25, and 12.5 μM of kahweol or 10 μg/mL of retinol for a further 12 h. The activity of luciferase expressed was determined by a luminometer, as reported previously [43].

### 4.7. Evaluation of Messenger RNA Levels through Reverse Transcriptase PCR

To analyze messenger RNA expression, HaCaT cells were plated in six-well plates with a 2.0 × 10^5^ cells/mL cell density. HaCaT cells were treated with 3.125, 6.25, and 12.5 μM of kahweol for 6, 9, 12, and 24 h. After discarding the supernatant, total RNA was isolated with TRIzol reagent. The PCR reaction was conducted under the following conditions: five minutes at 98 °C for preliminary denaturation, 15 s at 98 °C for denaturation, 15 s at 56 °C to 61 °C for annealing, one minute at 72 °C for extension, and five minutes at 72 °C for the last extension, as reported previously [44]. The lists for the primer sequences, annealing temperatures, and running cycles may be found in Table 1 and Table 2, respectively.

### 4.8. Total Cell Lysate Preparation

HaCaT cells were plated onto a 35 mm culture plate at 0.4 × 10^5^ cells/mL using fresh complete culture medium. Cells were treated with 12.5 μM of kahweol in different time points. Cells were collected by cold PBS. After adding lysis buffer, cells were incubated on ice for 15 min. Then, the mixture was spun down at 12,000 rpm for five minutes to obtain supernatant.

### 4.9. Immunoblotting

To prepare the loading sample, the absorbance of protein was detected at 570 nm using the spectrophotometer. Every loading sample contained 40 μg of proteins. Immunoblotting was performed as described previously [45,46].

### 4.10. Hyaluronan (HA) Quantification

To determine HA secretion, supernatant media was filtered by 0.2 μM syringe filter. To detect HA contents, HaCaT cells were washed by PBS and then centrifuged to remove PBS and get dry cell pellet. 25 mM Borax/H_2_SO_4_ reagent was mixed with cell pellet and 15 μL filtered media, separately. After 5 min incubation in room temperature, 50 μL 0.1% Carbazole/EtOH reagent was added to each tube. After vortexing, samples were kept in 95 °C for 10 min and then kept at room temperature around 15 min to cool down. Reactant was distributed in 96-well plates with 100 μL per well, and the absorbance was detected at 550 nM.

### 4.11. Statistical Analysis

Mean ± standard deviation values were used to demonstrate the data carried out at least three samples in this research. For statistical comparisons, the results were analyzed using Mann–Whitney test. A *p*-value < 0.05 was considered statistically significant. All statistical tests were carried out using SPSS (version 26.0, 2019, IBM Corp., Armonk, NY, USA). Similar experimental data were also observed using an additional independent set of experiments conducted using the same numbers of samples.

## 5. Conclusions

**Figure 4:**
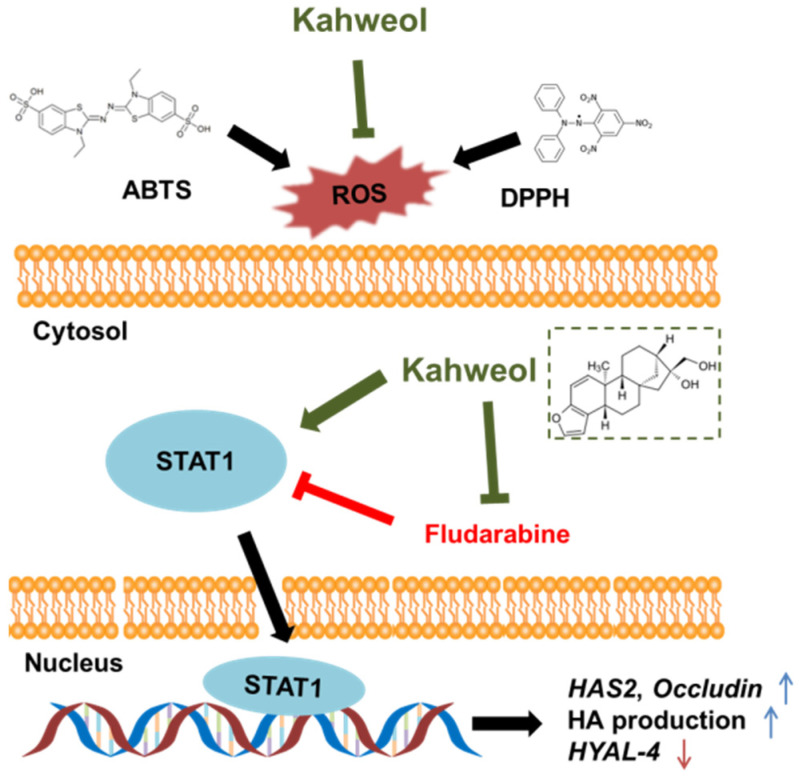
Schematic representation of molecules regulated by Kahweol.

In this research, we demonstrated that the promotion of the skin-moisturizing activities of kahweol was upregulated through STAT1 (Figure 4). Kahweol showed a good ability to enhance the expression levels of skin hydration, skin barrier, and skin moisture retention factors as well as HA production, implying that kahweol might be an effective component in skin-protective cosmetics.

## Figures and Tables

**Table 1:** Sequences of PCR primers used in this study.

Target	Direction	Sequences (5′ to 3′)
*HAS1*	Forward Reverse	CCACCCAGTACAGCGTCAAC CATGGTGCTTCTGTCGCTCT
*HAS2*	Forward Reverse	TGACAGGCATCTCACGAACC TGGCGGGAAGTAAACTCGAC
*TGM-1*	Forward Reverse	GAAATGCGGCAGATGACGAC AACTCCCCAGCGTCTGATTG
*Claudin*	Forward Reverse	TTGGGCTTCATTCTCGCCTT GAGGATGCCAACCACCATCA
*Occludin*	Forward Reverse	GGAGTGAACCCAACTGCTCA CCTGGGGATCCACAACACAG
*Involucrin*	Forward Reverse	TCCTCCTCCAGTCAATACCCA TGCTCAGGCAGTCCCTTTAC
*HYAL-4*	Forward Reverse	TGAGCTCTCTTGGCTCTGGA AGGCAGCACTTTCTCCTATGG
*GAPDH*	Forward Reverse	GGTCACCAGGGCTGCTTTTA GATGGCATGGACTGTGGTCA

**Table 2:** Annealing temperatures and running cycles adopted in this study.

Name	Annealing Temperature (°C)	Running Cycle
*HAS1*, *TGM-1*	56	38
*HAS2*	61	38
*occludin*, *claudin*, *involucrin*	56	35
*HYAL-4*	58	34
*GAPDH*	55	34

## Data Availability

The data used to support the findings of this study are available from the corresponding author upon request.

## Abbreviations

AP-1	Activator protein 1
HA	hyaluronic acid
TGM-1	transglutaminase 1
HAS	HA synthase
HYALs	hyaluronidases
MTT	3-4-5-dimethylthiazol-2-yl)-2-5-diphenyltetrazolium bromide
ABTS	2,20-azino-bis (3ethylbenzothiazoline-6-sulphonic acid) diammonium salt
DPPH	diphenyl-2-picryl-hydrazyl
AA	ascorbic acid
